# Voriconazole treatment of *Candida tropicalis* meningitis: persistence of (1,3)-β-d-glucan in the cerebrospinal fluid is a marker of clinical and microbiological failure: A case report

**DOI:** 10.1097/MD.0000000000004474

**Published:** 2016-08-07

**Authors:** Giancarlo Ceccarelli, Maria Cristina Ghezzi, Giammarco Raponi, Grazia Brunetti, Carolina Marsiglia, Stefania Fallani, Andrea Novelli, Mario Venditti

**Affiliations:** a Department of Public Health and Infectious Diseases. University of Rome “Sapienza”, Azienda Policlinico Umberto I, Rome; b Department of Health Sciences (DSS), Section of Clinical Pharmacology and Oncology, Università degli Studi, Florence, Italy.

**Keywords:** 1,3-β-d-glucan, biofilm, *Candida tropicalis*, case report, cerebrospinal fluid shunt, meningitis, voriconazole

## Abstract

**Introduction:**

Infections are still the most common complications of cerebral shunt procedures. Even though fungal etiologies are considered to be rare, they are associated with significant morbidity and mortality. Due to their uncommonness, diagnostic procedures and optimal therapy are poorly defined. We report a case of Candida tropicalis infection of ventriculo-peritoneal cerebrospinal fluid (CSF) shunt in a 49-year-old immune competent male treated with voriconazole (VOR).

**Methods:**

Microbiological and CSF markers (1,3-b-D-glucan-BDG) of fungal infection, biofilm production capacity, sensitivity of serial isolates of the pathogen, and the concentration of the antifungal drug have been monitored and related to the clinical course of this infection.

**Results:**

Despite appropriate treatment with VOR, in terms of adequate achieved CSF drug concentrations and initial effective therapeutic response, loss of VOR susceptibility of the C tropicalis and treatment failure were observed.

**Conclusion:**

Biofilm production of the C. tropicalis isolate might have had a significant role in treatment failure. Of interest, clinical and microbiological unfavorable outcome was anticipated by persistence of BDG in CSF. Rising titers of this marker were associated with relapse of fungal infection.

## 1 Introduction

*Candida* species are an uncommon cause of central nervous system (CNS) infections.^[1]^ Until now, few cases of *Candida tropicalis* infections of CNS have been reported in the medical literature.^[2,3]^ Therefore, risk factors, prognosis, diagnostic procedures, and optimal therapy are poorly defined. Despite appropriate therapy, the referred mortality of patients affected by *C tropicalis* meningitis approached 30%.^[1,2]^ We describe a case of an infection of the cerebrospinal fluid (CSF) shunt device in a middle-aged patient sustained by *C tropicalis*. Peculiarities of this report of such a rare infection are that measurement of (1,3)-β-d-Glucan (BDG) in the CSF, biofilm production capacity and modifications of the azole susceptibility of serial isolates of the pathogen, as well as antifungal drugs CSF concentration have been monitored and related to the clinical outcome.

## 2 Case report

**Figure 1:**
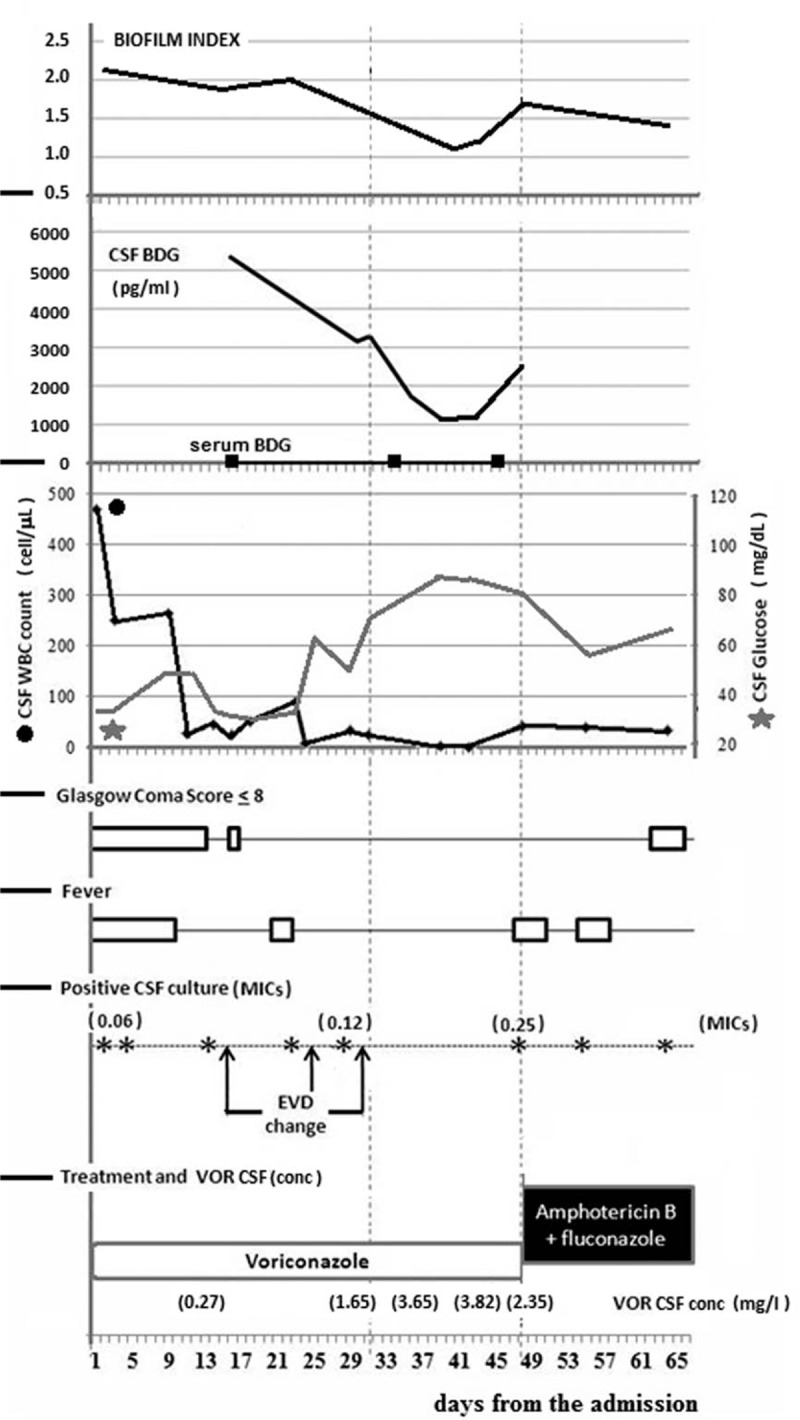
Clinical course, microbiological and laboratory monitoring of meningitis caused by *C tropicalis.* CSF BDG = cerebrospinal fluid (1,3)-β-d-Glucan, CSF WBC = cerebrospinal fluid white blood cells, CSF = *c*erebrospinal fluid, EDV = external ventricular drainage, MICs = minimal inhibitory concentrations, VOR BDG (conc) = voriconazole concentrations in cerebrospinal fluid).

A 49-year-old immune-competent male was admitted to the Neurosurgical Intensive Care for fever (up to 38°C) associated with ideomotor slowdown, severe confusional state, and meningism. Two months prior hospitalization, the patient underwent surgical removal of a *pontocerebellar* acoustic neuroma with placement of a ventriculo-peritoneal (VP) shunt and received an successful 10-day Fluconazole (FLU) treatment course for a catheter-related *C tropicalis* urinary tract infection. CSF analysis, performed at the admission, revealed glucose concentration of 34.2 mg/dL, protein level of 332 mg/dL, and leukocytes cell count of 471 cells/μL (75% polymorphonucleocytes). No signs of persistent *Candida* urinary tract infection were found in serial urine analysis and cultures performed during this second hospitalization. Fungal meningitis was diagnosed on the basis of *C tropicalis* isolation from the *CSF culture*. The same pathogen was also cultured from the removed VP *shunt*, while blood cultures were negative. Intravenous voriconazole (VOR) was started (loading dose 6 mg/Kg/q12 for 2 doses, then 4 mg/Kg/q12) and an external ventricular drainage (EVD) was inserted. The clinical course and the main laboratory findings, expressed in function of time, are shown in Fig. 1.

On day 13, 22, and 29 of VOR treatment, *C tropicalis* was still cultured from CSF and consequently EVD was changed. On the day 31 of *VOR treatment,* CSF cell count and glucose concentration achieved normal values and CSF culture yielded no microbial growth. With apparent *C tropicalis* microbial eradication from CSF, a clinical improvement of patient's condition in terms of Glascow Coma Score (Fig. 1) has been observed with initial impressive reduction of CSF BDG.

*C tropicalis* was isolated again from the CSF culture on the day 48 of therapy concurrently with new clinical-neurological deterioration. Increased CSF BDG levels proceeded new isolation of the offending pathogen from CSF. No increase in serum BDG has been detected in day 16, 34, 45 of VOR treatment (Fig. 1).

*VOR* was replaced by liposomal Amphotericin B (*LAMB*) (3 mg/kg/daily) along with FLU (loading dose 800 mg, then 400 mg/daily) and EVD was changed again. On the day 66 of antifungal therapy, the patient died for a concurrent bacterial sepsis, showing no further significant clinical improvement of his meningitis after the replacement of VOR by LAMB along with FLU. CSF cultures remained positive for *C tropicalis*. Given that interventions and all investigations described in this case report were performed as part of the standard health care whose informed consent was obtained, no ethical approval was either sought or obliged.

## 3 Microbiological and laboratory studies

### 3.1 *C tropicalis* susceptibility testing

The antifungal *susceptibility* of *C tropicalis* in CSF samples was tested by means of the minimal inhibitory concentrations (MICs) measurement (Sensititre Yeast One 10; Trek Diagnostic Systems Thermo Fisher Scientific, Rodano (MI), Italy), according to CLSI M27A3 guidelines.^[4]^ The s*usceptibility* of the *C tropicalis* isolates was monitored and the changes recorded over the time (Table 1). A gradual rise (up to 2 log_2_ dilution factor) of the *VOR* MICs was observed in serial CSF *C tropicalis* isolates**.**

**Table 1:**
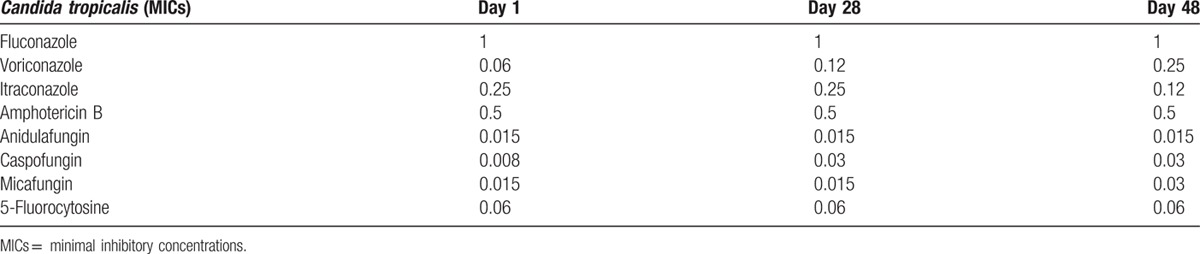
Changes of s*usceptibility* of *C tropicalis* isolates *tested* were reported in function of the time.

### 3.2 C tropicalis biofilm production

Biofilm production by *C tropicalis* strains was measured through XTT-reduction dehydrogenase assay and expressed as the ratio of the mean sample absorbance performed in triplicate experiments, over the 50% mean absorbance of a reference *C. albicans* strain (SA40, kind gift of F. De Bernardis, National Health Institute), in terms of biofilm index (BFI).^[5,6]^ Clinical isolates with BFI ≥0.5 were considered biofilm-producing strains. The aim of this experiment was to determine eventual time-related changes in biofilm-forming ability of strains of *C tropicalis* isolated from the CSF samples. All the isolates of *C tropicalis* were biofilm producers. The BFI fell from 2.1 to 1.1, with a 52% reduction at day 40. The recurrence of the isolation of *C tropicalis* on the day 48 was accompanied by a rise in the BFI (1.6 and 1.4 at days 48 and 52, respectively).

### 3.3 CSF (1,3)-β-d-glucan measurement

CSF BDG measurement was performed by a method based upon a modification of the *Limulus amebocyte* lysate pathway, using a commercially available diagnostic kit (Fungitell; Cape Cod Inc., East Falmouth, MA, USA), according to the manufacturer instructions. Glucan-free dilution tubes and pipette tips were used, and all assays were performed in a biosafety cabinet that had not been used to manipulate fungal cultures. Individuals performing the assay were blinded to all diagnostic and clinical information. This analysis was performed posthumously on CSF samples stored in the biobank. Our results showed a progressive decrease in the levels of CSF BDG (from 5230 to 1233 pg/mL), even though the values were constantly high and the negativity of the marker was never reached. Of note was a new gradual rise in the BDG in the CSF samples some days before the new isolation of *C tropicalis* (Fig. 1).

### 3.4 Voriconazole CSF concentrations

VOR plasma concentration was determined by high-performance liquid chromatography (HPLC), using Perkin Elmer chromatograph (Series 200, Beaconsfield, Perkin Elmer, USA) with wavelength ultraviolet (UV) detector set at 255 nm. An aliquot (0.5 mL) was pipetted into a 2 mL polypropylene tube (Sarstedt, Leicester, UK) and acetonitrile (1.0 mL) added. The mixture was vortex mixed briefly and after standing for 10 minutes at room temperature the mixture was centrifuged at 1200*g* for 10 minutes. Then, the supernatant was transferred in polypropylene autosampler vial and 0.180 mL injected into the HPLC system. This assay was linear (*r* = 0.9997) over the concentration of 0.1 to 50 mg/L. Validation data for accuracy and precision were coefficient of variation between 2.4% and 7.3%; intra-day accuracy was in the range of 95% to 106.5 %.^[7]^

A median CSF VOR concentration of 2 mg/L (range 0.27–3.82 mg/L) was registered, while the mean blood level of VOR was 2.48 mg/L (range 1.91–3.05 mg/L). The CSF/plasma VOR concentration ratio was 0.8. The CSF VOR concentrations observed were always higher than the coeval MIC of *C tropicalis*.

## 4 Discussion

The Italian Consensus for invasive candidiasis management (ITALIC) has suggested that *VOR* should be used as a first-line treatment for CNS *Candida* infections.^[8]^ The rationale for the use of this drug in this setting is linked to its ability to reach high concentrations in the CSF.^[9–11]^ Lutsar et al reported that in patients treated with *VOR* for CNS fungal infections, the median CSF/plasma ratio was 0.46 (range 0.22–1.0).^[9]^ Wiederhold et al,^[10]^ testing 173 CSF samples, confirmed a similar result (CSF/plasma ratio 0.52) and described that the median quantifiable CSF level of *VOR* was 2.47 mg/L (range 0–15.3 mg/L) with CSF concentrations more heavily distributed toward the lower end of the range. However, the CSF concentration and the ratio may vary depending on the serum *drug* concentrations *and* is influenced by several variables such as liver disease, age, genetic polymorphism of the cytochrome CYP2C19, and comedications.^[11]^ These variables may lead to large inter- and intra-patient differences in *VOR* pharmacokinetic, perhaps sometimes associated with a decreased efficacy. Therefore, therapeutic drug monitoring (TDM) is required to improve the efficacy and the safety of VOR therapy in patients with invasive mycoses.

VOR was used in our case and the drug achieved plasma and CSF levels that should have provided a clinical efficacy (CSF/plasma concentration ratio of 0.8). Furthermore, the MICs of *C tropicalis* isolates, ranging from 0.06 to 0.25 mg/L, were always below the resistance breakpoint that is >0.5 mg/l, according to the CLSI interpretation.^[12]^ However, a progressive increase of the MIC values was observed during the course of VOR treatment and a value of 0.25 mg/L was observed on day 48 of VOR therapy. To date, according to new CLSI VOR clinical breakpoints for *C tropicalis* (available only after the occurrence of the described case), MICs values for VOR of 0.25 to 0.5 mg/L should be interpreted as intermediate sensitivity.^[12]^ Of interest, a recent Italian study reported that 99% of the isolates of *C tropicalis* tested were highly susceptible to VOR (0.008 mg/L≤ MICs ≤0.25 mg/L; MIC_50_ 0.03 mg/L; and MIC_90_ 0.06 mg/L) with only 1 out of 102 isolates showing dose-dependent susceptibility.^[13]^ These data were confirmed by the results of a large multicenter study conducted in Europe and North and South America where only 2.2% and 3.5% of 3127 *C tropicalis* isolates showed MICs for VOR of 0.25 and ≥0.5 mg/L, respectively.^[14]^ As a matter of fact, in our case, we cannot exclude that the above-mentioned loss in susceptibility to VOR might have affected the efficacy of therapy.

Following *VOR treatment failure*, LAMB as well as FLU therapy was attempted on the basis of previous apparently favorable in vivo and in vitro observations of this antifungal agents combination.^[15,16]^ However, the efficacy of this salvage therapy could not be fully evaluated, as the patient finally died for bacterial superinfection.

Further insights might be gained by the observation of biofilm production by all consecutive fungal isolates. Indeed, the ability of *C tropicalis* to form biofilm on devices is considered a potential virulence factor and might have represented another limitation for therapy with VOR (or LAMB) in our case.^[17,18]^ Such a phenomenon might be strongly implicated in the final VOR treatment failure despite susceptibility of fungal isolates and prompt removal and substitution of EVDs.

Of interest, as shown in Fig. 1, a progressive reduction of *BFI* has been observed in serial isolates during antifungal therapy. To this end, recent studies showed that VOR fails to eradicate *Candida spp.* in mature biofilm, but seems to display in vitro efficacy in the reduction of biofilm formation.^[19–21]^ Of course, knowledge of the clinical significance of these observations requires for further investigations.

The therapeutic efficacy of VOR was initially assumed by the progressive restoration of the normal physical-chemical and microbiological CSF parameters. These findings were also indirectly confirmed by the gradual decline of CSF BDG observed until a few days before the recurrence of positive CSF culture under VOR therapy. Erythrocyte sedimentation rate (ESR), C reactive protein (CRP), and BDG might be used for monitoring efficacy of antifungal therapy. Nevertheless, ESR and CRP are nonspecific inflammatory markers and can be affected by concomitant conditions such as concurrent bacterial infections that compromise their usefulness in monitoring the fungal disease in critically ill patients, as it occurred in our patients.^[22]^ Therefore, due to these possible bias, serum and/or BDG seem to be a suitable marker in this setting.^[23]^

Recent studies, performed in animal models and on human beings, proposed *CSF* BDG as a marker for the detection of fungal infections in the CNS and for clinical therapy monitoring.^[24–28]^ In our case, during VOR treatment, the retrospective analysis in serial samples of CSF demonstrated persistence of BDG, even when cultures yielded no microbial growth, albeit with a progressive reduction of titers. On the basis of these observations, persistence of BDG in the CSF might suggest the need for maintaining the antifungal treatment despite negative cultures. On the contrary, clearance of this marker might suggest the optimal timing to reposition a VP shunt. Not less important, a sudden increase of CSF BDG values during antifungal therapy, as that observed in our case before the *C tropicalis* recurrence, might represent a warning sign for the treatment failure.

On the contrary, serum BDG levels do not seem to be concordant with CSF BDG levels: in fact in our case, BDG values were significantly higher in CSF than in blood. As previously suggested, this gap could suggest isolated CNS infection without significant spillover into venous blood or that BDG may be more rapidly cleared in blood than in CSF.^[26]^ According with the results of the fungal meningitis rabbit model proposed by Petraitiene et al,^[29]^ plasmatic levels of β-glucan lower than CSF levels hint a compartmentalization of this polymeric carbohydrate, with higher concentrations within the CNS not readily crossing the blood–brain barrier. Taken together, these data could indicate that serum BDG values may not be helpful in determining invasion of the CNS by a fungal pathogen.

Finally, despite the data supporting the efficacy of VOR in CNS fungal infections, use of this azole as first-line therapy in CSF *Candida* infections, as proposed by ITALIC,^[8]^ is not shared by the most recent guidelines for the “Management of Candidiasis,” updated by the Infectious Diseases Society of America (IDSA) in 2016.^[12]^ In these recommendations, VOR is not cited as a possible choice in the treatment of CSN *Candida* infections, although it is recognized that it can achieve significant CSF concentration, potentially active against susceptible isolates, and it has been shown to be efficacious in small case series.^[8–10,12]^ Wide variability in serum levels, need of the TDM, common drug–drug interactions with potential toxicity might have discouraged the use of VOR in these Guidelines.

## 5 Conclusions

We described a rare case of *C tropicalis* meningitis wherein appropriate treatment with VOR, in terms of adequate achieved CSF drug concentrations, was associated with loss of VOR susceptibility of the offending pathogen and treatment failure. Of interest, clinical and microbiological unfavorable outcome was anticipated by persistence of BDG in CSF. Eventually, rising titers of this marker were associated with clinical and microbiological relapse of fungal infection. Biofilm production of the *C tropicalis* isolate might have had a significant role in treatment failure. The importance of all these data should be confirmed by further studies.
